# Projected heat-related mortality under climate change in the metropolitan area of Skopje

**DOI:** 10.1186/s12889-016-3077-y

**Published:** 2016-05-16

**Authors:** Gerardo Sanchez Martinez, Michela Baccini, Koen De Ridder, Hans Hooyberghs, Wouter Lefebvre, Vladimir Kendrovski, Kristen Scott, Margarita Spasenovska

**Affiliations:** WHO European Centre for Environment and Health, WHO Regional Office for Europe, Platz der Vereinten Nationen 1, 53113 Bonn, Germany; Department of Statistics, Informatics and Applications (DiSIA), University of Florence, Viale Morgagni 59, 50134 Florence, Italy; Biostatistics Unit, Cancer Prevention and Research Institute (ISPO), Via Cosimo Il Vecchio 2, 50139 Florence, Italy; VITO (Flemish Institute for Technological Research), Urban Climate Team, Boeretang 200, 2400 Mol, Belgium; WHO Country Office, the former Yugoslav Republic of Macedonia, Drezdenska 22, 1000 Skopje, Macedonia

**Keywords:** Heat waves, Skopje, Heat-related mortality, Climate change

## Abstract

**Background:**

Excessive summer heat is a serious environmental health problem in Skopje, the capital and largest city of the former Yugoslav Republic of Macedonia. This paper attempts to forecast the impact of heat on mortality in Skopje in two future periods under climate change and compare it with a historical baseline period.

**Methods:**

After ascertaining the relationship between daily mean ambient air temperature and daily mortality in Skopje, we modelled the evolution of ambient temperatures in the city under a Representative Concentration Pathway scenario (RCP8.5) and the evolution of the city population in two future time periods: 2026–2045 and 2081–2100, and in a past time period (1986–2005) to serve as baseline for comparison. We then calculated the projected average annual mortality attributable to heat in the absence of adaptation or acclimatization during those time windows, and evaluated the contribution of each source of uncertainty on the final impact.

**Results:**

Our estimates suggest that, compared to the baseline period (1986–2005), heat-related mortality in Skopje would more than double in 2026–2045, and more than quadruple in 2081–2100. When considering the impact in 2081–2100, sampling variability around the heat–mortality relationship and climate model explained 40.3 and 46.6 % of total variability.

**Conclusion:**

These results highlight the importance of a long-term perspective in the public health prevention of heat exposure, particularly in the context of a changing climate.

**Electronic supplementary material:**

The online version of this article (doi:10.1186/s12889-016-3077-y) contains supplementary material, which is available to authorized users.

## Background

The epidemiologic evidence on the association between heat and health impacts in major cities of western Europe is robust [1–4], especially concerning the observed increase in mortality and the risk factors that increase population vulnerability [5, 6]. Exposure to environmental heat is potentially larger in urban agglomerations [7, 8] due to the comparative abundance of heat-absorbing surfaces and materials, a phenomenon known as “urban heat island” effect [9]. Other factors, like household insulation and access to air conditioning, socioeconomic factors, and individual vulnerability, may exacerbate heat-related risks for some specific urban populations or areas [6, 10, 11]. The Intergovernmental Panel on Climate Change (IPCC) [12] considers it *likely* that climate change-driven increases in daily maximum temperatures may have already increased the number of heat-related deaths. This hypothesis has not been conclusively proven; a recent study found a decrease in the relative risk for heat-related mortality in 2006 compared with 1993 in several countries [13]. However, climate change scenarios almost unanimously project rising temperatures and an increase in frequency and intensity of heat waves globally and in the European Region [14]. Since it is unclear how much the resulting health impacts might be minimized due to acclimatization [15–19], an increase in heat-related adverse health effects, in the absence of adaptation, may follow [20–22].

Baccini et al. [23] studied the relationship between increased maximum apparent temperatures and mortality in 15 European cities, finding an increase in mortality of 3.1 % (95 % CI: 0.6–5.7) in Mediterranean cities and an increase of 1.8 % (95 % CI: 0.1–3.6) in north-continental cities for every 1 °C increase in maximum apparent temperature above a city-specific threshold. An additional study, conducted across 12 European cities, found a positive, but variable, association between temperature and hospital admissions for respiratory disorders [2]. For each 1 °C increase above a city-specific threshold in maximum apparent temperature, respiratory admissions increased by up to 4.5 % in Mediterranean cities compared to 3.1 % in north-continental cities.

In contrast with the solid evidence base for the association between heat and health in some western European countries, research is scarce in south-eastern European countries, particularly those outside the EU. There is, however, some evidence on the association between heat and health from urban settings in Serbia and the former Yugoslav Republic of Macedonia. Stanojevic et al. [24] estimated that 253 persons died due to heat waves in Belgrade during the summer of 2007, during which temperature records were broken in many monitoring stations in in the country. In the case of the former Yugoslav Republic of Macedonia, the summer heat waves of 2007 resulted in more than 1000 excess deaths at the national level [25]. Intensity and duration of heat waves were the two main factors predicting episodes of excess mortality in Skopje and Belgrade, and both of these coincided during the heat waves in summer 2007 making that summer the most severe in the researched period [25].

The direct health effects of heat are already a significant problem in the former Yugoslav Republic of Macedonia, and could worsen further under climate change, especially with increasing urbanization and warming prospects for the subregion [26]. In response to this threat, the national government endorsed a heat–health action plan based on a defined set of actions to protect health from a heat wave [27]. The plan, designed for current conditions like others within the subregion and elsewhere, was first tested throughout the country in the summer of 2010, and fully implemented in the following summers.

This paper attempts to describe the change in environmental heat conditions that the urban population of Skopje could experience under climate change, and the effects such change could have on excess heat-related mortality in two future periods within the 21^st^ century.

## Methods

### Urban Climate assessment

#### The UrbClim model

In this study, the current and the future urban climate in Skopje was simulated through the UrbClim model, an urban climate model designed to study the urban heat island effect (UHI) at a spatial resolution of a few hundred metres, for a typical domain size of 20 km by 20 km. The model scales down large-scale weather conditions to agglomeration-scale and computes the impact of urban development on the most important weather parameters, such as temperature and humidity. UrbClim is composed of a land surface scheme describing the physics of energy and water exchange between the soil and the atmosphere in the city, coupled to a three-dimensional boundary layer module, which models the atmosphere above the urban agglomeration. The synoptic (large-scale) atmospheric conditions are taken from global model output fields. Local terrain and surface data influence the heat fluxes and evaporation within the urban boundaries. A detailed description of the model is provided in De Ridder [28]. The model has been subjected to exhaustive validation; within the scope of the European RAMSES and NACLIM projects, model results have been compared with hourly temperature measurements for, amongst others, London (United Kingdom), Bilbao (Spain), Antwerp (Belgium), Berlin (Germany), Almada (Portugal) and Paris (France) [9, 28, 29].

The terrain input for the current study consists of the spatial distribution of land use types, the degree of covering of the soil by artificial structures such as buildings and roads, and the vegetation cover fraction with a spatial resolution of 250 metres. These quantities were taken from publicly available data sets, specifically, the 2006 CORINE Land Cover data for Europe, the European Environment Agency soil sealing data, and the Normalized Difference Vegetation Index (NDVI) acquired by the MODIS instrument on the TERRA satellite, respectively.

#### Current and future climate assessment

The current urban climate of Skopje was simulated by coupling UrbClim to large-scale meteorological data of the European Centre for Medium-Range Weather Forecasts (ECMWF): for the period 1986–2012 the model was coupled to ERA-Interim reanalysis data of the ECMWF. The results are hourly temperature maps of Skopje with a resolution of 250 metres. In the mortality analysis and in the impact assessment, a single urban mean temperature was considered for each day, obtained by calculating the mean of the daily mean temperatures of all the grid points within the city boundaries of Skopje.

To assess the future urban climate of Skopje, UrbClim was coupled to the output of global climate models (GCMs) contained in the Coupled Model Intercomparison Project 5 (CMIP5) archive of the Intergovernmental Panel on Climate Change (IPCC). The details of the coupling are described in [30], so here we only highlight the points important for the assessment at hand. By coupling UrbClim to the output of the GCMs, we obtain projections for the urban heat island in Skopje in the future. Note that the methodology at hand considers the urban form to be constant over time. As such, the results for the future periods in the remainder of the paper refer to an unchanging and unadapted city.

The IPCC fifth assessment report (AR5) [14] identifies four Representative Concentration Pathway climate scenarios, ranging from very strong (RCP2.6) to weak mitigation scenarios (RCP8.5). Within this study, we employed the RCP8.5 scenario: although this is the scenario with the largest warming potential, current emission trends continue to track along the trends of this scenario [31].

The UrbClim model requires forcing by an ensemble of GCM outputs in order to properly represent the uncertainty associated with the global climate projections. That allows obtaining mean values, tendencies and an uncertainty range. The IPCC AR5 endorses this as good practice, highlighting that the agreement between ensemble means and climate data exceeds the agreement with any single climate model by a large amount [14, 32]. Based on data requirements of the UrbClim model and data availability, 11 GCMs were selected (see [30] for an overview of these models). The IPCC also identifies three timeframes: a baseline period (1986–2005), a near future period (2026–2045) and an end-of-century time period (2081–2100) [14]. We used the same time frames in our simulations. Note that, for the urban climate projections, the ERA-interim runs described above are considered as the benchmark for all future climate projections; we therefore introduce a bias correction which effectively reduces the difference between the ERA-Interim runs and the GCM runs for the baseline period [30].

The future urban climate in Skopje has been simulated for all 11 GCMs [30]. However, due to computational limitations, only three of the 11 climate models were used for assessing future impact of heat on population health in Skopje: MRI-CGCM3 [33], IPSL-CM5A-MR [34] and GISS-E2-R [35]. These models correspond respectively to the models that, of all 11 considered models, exhibit the lowest, the maximal and the median mean temperature for the far future period (2081–2100). Hence, this selection of three GCMs mimics the spread of the complete set of 11 GCMs.

The outcomes of the simulations were hourly temperature maps of Skopje with a resolution of 250 metres. We obtained maps for the three different GCMs (MRI-CGCM3, IPSL-CM5A-MR and GISS-E2-R) for the three different time periods (1986–2005, 2026–2045 and 2081–2100). Then the urban daily mean temperature was obtained by calculating the average of the daily mean temperatures of the grid cells within the city boundaries of Skopje. These urban temperatures are hereafter used in the health impact assessment.

### Health impact assessment

The geographical area under study comprises the 10 districts within the municipality of Skopje (not including Sopište, physically segregated from the Skopje agglomeration). With the aim to assess the impact of heat on mortality in this area during the baseline period 1986–2005, the near future period 2026–2045 and the far future period 2081–2100 under RCP8.5, we first focused on the estimation of the heat–mortality relationship, to be successively used for health impact assessment, based on the historical data from a sample period, comprising the years 2007–2011, and on the current estimates and predictions of the future population size. Then we combined these results with the meteorological projections for the three time periods to calculate the impact of heat in terms of attributable deaths (AD). In the following sections we describe the statistical models used for the analysis and their underlying assumptions.

#### Population data and projections

Taking as reference the population data for the Skopje agglomeration in 2002 (the year of the latest available census in the former Yugoslav Republic of Macedonia) and a recent estimate of the population 10 years later, in 2012, obtained from the country’s statistical office, we considered two different models to predict future population sizes in Skopje: an exponential model and a logistic model [36]. The exponential model assumes a yearly increase of the population according to a constant growth rate. It provides reliable short-term predictions (10–20 years), but its use for long-term predictions may not be optimal, so we also used a logistic model, assuming various limits for the population size through a “carrying capacity” parameter *K* – see the “Additional file 1” section for details on models specification.

#### Heat–mortality relationship

In order to estimate the heat–mortality relationship to be successively used for health impact assessment, we collected daily number of deaths from all causes. The complete daily mortality dataset was obtained from the country’s Institute of Public Health. In order to assure homogeneity between historical and future predicted meteorological data, daily temperature and humidity data for the sample period 2007–2011 were obtained by coupling the UrbClim model to the ERA-interim reanalysis data of ECMWF large-scale meteorological data, as discussed in section Current and future climate assessment. In addition, daily concentrations of particulate matter with diameter <10 μm (PM_10_) for the city of Skopje were obtained from the country’s Ministry of the Environment for the sample period 2007–2011. The dataset used for the health impact assessment is available in the Additional file 2.

For validation of the model-generated series of temperature data, and in the absence of historical intra-urban data series of temperature measured within Skopje, we obtained hourly temperature time series measured at the Skopje airport through the online portal of the US National Climatic Data Center (NCDC, https://www.ncdc.noaa.gov/). Although this location lies just outside the city domain of the UrbClim model, the measurements can still be used to validate the rural temperatures around Skopje modelled by UrbClim. When considering the warm seasons (1 May–30 September, which corresponds with the dates of operation of the country’s heat wave early-warning system), these daily data were strongly correlated with those measured at the airport. For the summer of 2007 we obtained, for daily mean temperatures, a Pearson correlation coefficient of 0.96, and a bias of 0.5 degrees Celsius (with the UrbClim results being slightly warmer than the measurements). Similar results were obtained for the other years in the period 2007 – 2011. The magnitude of the differences is not related to the heat level, as similar correlations and biases are obtained when only the 20 % warmest or 20 % coolest summer days are taken into account. Although the correlations and bias for the mean temperatures indicate a very good comparison between the model and measured data, there are larger biases for daily minimal and maximal temperature. However, since the mortality analysis makes use of the mean daily values, these deviations in the daily pattern are of less importance.

Following the statistical approach proposed by Baccini et al. [23], we focused only on the warm seasons (1 May–30 September), so data consisted of five disjointed five-month daily time series. The outcome variable was the daily death count with assumed Poisson distribution. After assuming independence between summers and a first-order autocorrelation structure for the daily death counts within the same summer period, the analysis was based on a generalized estimating equations (GEE) approach, without robust adjustment of the standard errors, as recommended in the presence of few large clusters [37]. We included dummy variables in the models to account for holidays, day of the week and calendar month; linear and quadratic terms to pick up the yearly long-term time trend; linear and quadratic terms for dew point; linear term for the average of the current and the previous day concentration of PM_10_. However, since the need for adjusting for air pollution is arguable [38], a sensitivity analysis was conducted by removing this last variable from the model.

The literature does not provide clear indication on which is the best temperature indicator to investigate the effect of heat on mortality. Moreover, due to the strong correlation between different indicators, comparable results can be expected from using different indexes, for example daily mean or daily maximum temperature [39, 40]. Here, we focused on daily mean temperature. In particular, we studied the immediate effect of heat, considering as exposure indicator lag 0–3 days of mean temperature, defined as the average of the mean temperature in the current day and in the previous 3 days. With the aim to allow comparison with the literature [23, 41, 42] we repeated the analysis using as exposure indicator lag 0–3 days of maximum apparent temperature [43, 44].

First, we used a flexible parametric approach to describe the exposure–response curve, including a cubic regression spline in the model with five degrees of freedom for the exposure (the knots being placed on the quantiles of the exposure distribution). Second, we described the relationship between lag 0–3 days of mean temperature and mortality by two linear terms constrained to join at a common point, which we call threshold. The threshold estimate was obtained through the maximum likelihood approach proposed by Muggeo [45]. The final model for the number of daily deaths was the following:

1 $$ \begin{array}{l}{y}_i\sim Poisson\left({\lambda}_i\right)\\ {} log\left({\lambda}_i\right)=\alpha + confounders+{\beta}_1\times \left({T}_i-{T}_0\right)\times I\left({T}_i\le {T}_0\right)+{\beta}_2\times \left({T}_i-{T}_0\right)\kern0.5em \times I\left({T}_i>{T}_0\right)\end{array} $$

where:

T_*i*_ was the exposure (lag 0–3 mean temperature) in day *i*,

T_0_ was the threshold,

I(T_*i*_ > T_0_) was an indicator function equal to 1 if T_*i*_ > T_0_ and 0 elsewhere and I(T_*i*_ ≤ T_0_) was an indicator function equal to 1 if T_*i*_ ≤ T_0_ and 0 elsewhere,

β_1_ and β_2_ were the linear coefficients expressing on a log scale the effect of 1 °C increase in exposure under and above the threshold, respectively.

#### Attributable deaths calculation

According to model (1), the fraction of deaths attributable to mean temperature (Attributable Fraction, AF) above the threshold T_0_ in day *i* is:

2 $$ A{F}_i=1-1/ exp\left[{\beta}_2 \times \left({T}_i-{T}_0\right) \times I\left({T}_i>{T}_0\right)\right] $$

If the number of daily deaths Y_*i*_ is known, the total number of attributable deaths (AD) during the period of interest can be calculated as sum of daily AD:

3 $$ AD={\displaystyle \sum_i}{Y}_iA{F}_i $$

If Y_*i*_ is unknown, the expected number of attributable deaths (AD) during the warm season for a specific year can be approximated by the following product:

4 $$ AD=P \times r \times \gamma \times {\displaystyle \sum_{i=1}^n}A{F}_i/n $$

where:

P is the observed population or the projected population for the year of interest,

r is the crude mortality rate,

γ is an estimate of the proportion of deaths during summer over the total annual number of deaths and

n is the number of warm season days (*n* = 153).

Equation (4) is valid if we can assume that the day by day correlation between number of deaths and AF is minimal; in other words, if we can assume that heat is not the main factor determining the daily mortality level.

The proportion γ of deaths during the warm season, estimated from the daily mortality time series of the sample period 2007–2011, was equal to 0.385. The crude mortality rate r that we used in the model was that for 2012, which was equal to 0.93 % (data from the country’s statistical office).

We used a Monte Carlo approach to account for sampling variability around the estimates of β_2_ and T_0_ [46]. We assumed a Normal distribution for both these parameters, with mean equal to the point estimate arising from the time series model and variance equal to the estimated variance. Then we independently sampled 10,000 values from these two Normal distributions and we used these values to calculate AD according to equations (2)–(4). In this way we obtained, for each climate model and each time period, a sample of 10,000 values from the AD distribution for each warm season of interest. We did not account for sampling variability around proportion γ and crude mortality rate r, which were considered known and constant over time

#### Variance-based sensitivity analysis

We calculated for each source of variability (sampling variability around the heat-mortality curve, inter-annual variability, climate model and population model), the relative contribution to the total variance of the average number of AD per year. We measured this contribution through the “total-effect index”, a quantity which measures the portion of total variance due to each source of uncertainty, including the variance caused by its interactions with all other sources [47]. Given a set of input variables X_1_, *X*_2_, …X_n_ and the output Y (AD per year in our specific application), the total variance index for the input X_*i*_ is defined as:

5 $$ {S}_{Ti}=\frac{E\left(Var\left(Y\Big|{X}_1,\dots {X}_{i-1},{X}_{i+1},\dots, {X}_n\right)\right)}{Var(Y)}=1-\frac{Var\left(E\left(Y\Big|{X}_1,\dots {X}_{i-1},{X}_{i+1},\dots, {X}_n\right)\right)}{Var(Y)} $$

We calculated the total variance indexes from the Monte Carlo simulations according to the previous equalities.

## Results

### Heat–mortality relationship for the sample period (2007–2011)

**Fig. 1:**
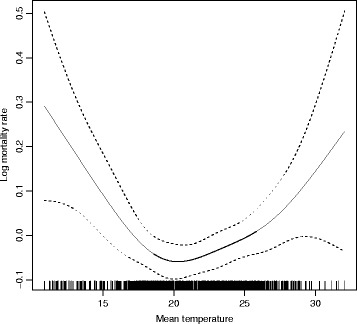
Adjusted relationship between lag 0–3 mean temperature and mortality for the period 2007–2011 during the warm season (1 May–30 September). Cubic regression spline (pointwise 95 % confidence bands) describing the relationship on a logarithmic scale

The average relationship between ambient temperatures and mortality in Skopje was ascertained for the sample period (2007–2011). This relationship can be described through an exposure–response curve. The non-parametric curve describing the relationship between lag 0–3 days of mean temperature and mortality in 2007–2011 is shown in Fig. 1. As expected, mortality decreases with temperature to a certain point, or threshold, beyond which it increases again. The relationship above the threshold is not far from linearity if we exclude very high exposures, for which, anyway, the confidence bands around the estimated curve are very large.

The maximum likelihood estimate of the threshold, i.e. the temperature associated with the minimum mortality rate, was 19.7 °C (90 % CI: 18.5; 20.9). Above this threshold, the estimated change in mortality associated with 1 °C increase in mean temperature was 1.70 % (90 % CI: 0.78; 2.64); under the threshold a reverse relation was found: decreasing exposure of 1 °C, mortality increased by 4.11 % (90 % CI: 2.33; 5.92). When considering maximum apparent temperature lag 0–3 days as exposure indicator; the shape of the curve was confirmed, but the minimum was around 30 °C and the change above the threshold was 4.47 % (90 % CI: 2.56; 6.42). After removing the air pollution term from the model, the threshold estimate did not change, but the percent increase above the threshold was slightly higher: 2.09 % (90 % CI: 1.28, 2.91), suggesting that by adjusting for PM_10_ we could have slightly underestimated the effect of mean temperature.

### Population predictions

**Fig. 2:**
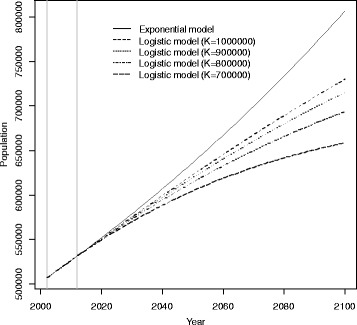
Projected population size for the city of Skopje from 2002 to 2100. Projections under exponential model and under logistic models with different values of the carrying capacity parameter (K). Vertical lines indicate the years 2002 and 2012

We obtained projections of the future population size under different models. The population growth from 2002 to 2100 under an exponential model and logistic models with different carrying capacity (K = 1,000,000; 900,000; 800,000; 700,000) are shown in Fig. 2. The vertical lines indicate the initial population (P_2002_) and the population after 10 years (P_2012_); these values were assumed as fixed and used to estimate the parameters of the population growth models. While the four models produced similar short-medium population size predictions, the expected number of inhabitants in 2100 tended to diverge in the long term.

### Attributable deaths

As a preliminary result, we obtained the impact of heat during the sample period 2007–2011. We estimated that during this period 4.5 % of deaths during the warm seasons were attributable to mean temperatures exceeding the estimated threshold of 19.7 °C, corresponding to 74 deaths per year (10^th^ and 90^th^ quantile: 52; 95). This result was obtained using the historical mortality and temperature data used to estimate the heat–mortality relationship and applying equations (2) and (3).

As explained, three different global climate models were used in the health impact assessment: MRI-CGCM3, IPSL-CM5A-MR and GISS-E2-R. These models correspond respectively to the models which, of all eleven considered models, exhibit the lowest, the maximal and the median temperature for the far future period (2081–2100). Henceforth, we refer to them as the minimal (MRI-CGCM3), the maximal (IPSL-CM5A-MR) and the median (GISS-E2-R) climate model. For all three models, daily mean temperatures for Skopje under RCP8.5 have been obtained for the baseline period (1986–2005), and the two future periods (2026–2045 and 2081–2100) [see section Current and future climate assessment]. Table 1 summarizes the mean and 95th percentile of the daily mean temperatures by time periods and climate model. Under the median climate model, the average of the daily mean temperatures is expected to be 22.9 °C in 2026–2045 and 26.1 °C in 2081–2100, compared with an average of 20.7 °C in the baseline period 1986–2005. Under the minimal model, the projected average is 21.6 °C in 2026–2045 and 24.6 °C in 2081–2100. Under the maximal model, the average in 2026–2045 is expected to be only slightly higher than under the median model, while it is expected to reach 28.5 °C in 2081–2100, with the 5 % of days having daily mean temperature larger than 37.7 °C.

**Table 1:** Mean (95th percentile) of the warm season (1 May–30 September) daily mean temperatures in degrees Celsius by time period and climate model

	Climate model		
Time period	Median	Minimal	Maximal
1986–2005	20.7 (27.4)		
2026–2045	22.9 (30.0)	21.6 (28.9)	22.9 (30.2)
2081–2100	26.1 (35.1)	24.6 (32.4)	28.5 (37.7)

Based on the series of daily mean temperatures for Skopje under RCP8.5, the projected AFs and the projected numbers of AD under different population models were calculated by applying equations (2) and (4). It should be noted that for the baseline period 1986–2005, the three climate models are calibrated to match the current climatic conditions described by the ERA-interim reanalysis data, so their values coincide and, as a consequence, AFs and AD overlap. Therefore, for the period 1986–2005, only the impacts obtained under the median climate model are presented.

Table 2 summarizes AD distributions obtained via Monte Carlo simulations in terms of their mean, 10^th^ and 90^th^percentiles. On average, during the baseline period (1986–2005) 3.3 % of deaths were attributable to temperatures above the threshold, for a total of 58 deaths per year. (10^th^ and 90^th^ quantile: 36; 85). This estimate is slightly lower than the estimate obtained for the sample period 2007–2011; this difference is likely due to the specific meteorological characteristics of the period 2007–2011, during which one important heat wave was observed in the Balkans, including the former Yugoslav Republic of Macedonia (in summer 2007) [25, 48].

**Table 2:** Estimated attributable fractions (AF), and mean, 10th percentile and 90th percentile of the distribution of the attributable deaths (AD) per year in Skopje for the time periods 1986–2005, 2026–2045 and 2081–2100, under different climate models and population growth models

	1986–2005	2026–2045			2081–2100		
	Climate model^a^	Climate model			Climate model		
	Median	Median	Minimal	Maximal	Median	Minimal	Maximal
*Average AF*	*3.3 %*	*5.8 %*	*4.7 %*	*6.6 %*	*9.8 %*	*8.1 %*	*13.2 %*
Population model	Attributable Deaths/year						
*Exponential*	58 (36; 85)	124 (84; 170)	100 (63; 143)	125 (80; 185)	272 (163; 388)	223 (148; 301)	366 (232; 512)
*Logistic K = 700,000*	55 (34; 81)	117 (80; 161)	95 (60; 135)	118 (75; 174)	226 (137; 321)	186 (123; 250)	304 (194; 422)
*Logistic K = 800,000*	55 (34; 81)	117 (80; 161)	95 (60; 135)	118 (75; 174)	235 (142; 334)	193 (128; 260)	316 (201; 439)
*Logistic K = 900,000*	55 (34; 81)	117 (80; 161)	95 (60; 135)	118 (75; 174)	240 (145; 341)	197 (131; 265)	323 (206; 449)
*Logistic K = 1,000,000*	55 (34; 81)	117 (80; 162)	95 (60; 136)	119 (76; 176)	244 (147; 346)	200 (132; 269)	327 (208; 456)

(^a^)For the baseline period 1986–2005, the three climate models are calibrated to match the current climatic conditions described by the ERA-interim reanalysis data, so their values coincide and, as a consequence, AFs and AD overlap. Therefore, for the period 1986–2005, only the impacts obtained under the median climate model are presented

AFs for the period 2026–2045 are expected to range from 4.7 % under the minimal climate model to 6.6 % under the maximal model. For the period 2081–2100, AFs are expected to range from 8.1 % to 13.2 %. Assuming an exponential model for population growth, heat is expected to be responsible on average for 124 deaths per year during the period 2026–2045 under the median climate model, and for 100 and 125 deaths under the minimum and the maximum climate models, respectively. Using logistic models for population prediction did not bring substantially different results.

For the period 2081–2100, under the exponential model for population growth, 272 AD per year are expected under the median climate model, 223 and 366 AD per year under the minimum and the maximum climate models, respectively. Using logistic models, lower predictions were obtained, with the number of AD decreasing as the carrying capacity parameter decreased.

For each combination of climate and population models, the inter-percentile range was very large, so that the intervals partially overlapped even when the maximum and the minimum climate models were compared. It should be noticed that reported inter-percentile ranges derive from sampling variability around threshold and slope and inter-annual variability of climate projections.

**Fig. 3:**
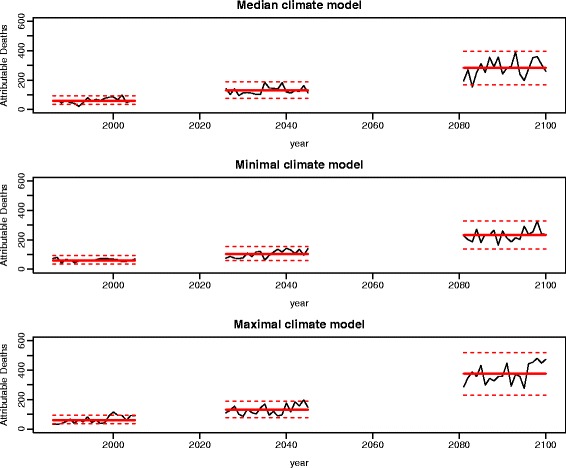
Attributable deaths predictions under the median, the minimal and the maximal climate models. An exponential model for population growth is assumed. Red lines indicate the average number of attributable deaths per year during each time period

Figure 3 shows AD predictions obtained for each year during the time laps of interest under the three climate models, after assuming an exponential model on population growth; red lines correspond to the average number of AD per year during the three time periods. A clear acceleration of heat impact over time is visible under the maximal climate model. Inter-annual variability of AD predictions appeared to be substantial, so that the lowest annual peaks under the maximal climate model and the highest annual peaks under the minimum climate model are expected to be close one to each other.

The total-effect indexes for each source of variability are reported in Table 3. When considering the baseline period, AD estimation involved only two sources of variability: sampling variability around threshold and slope, and inter-annual variability, contributing to 27.8 and 80.4 % of total variance, respectively. It should be noted that the total-effect indexes do not add up to 1, because they can include the common contribution of shared interactions [47]. When considering the period 2026–2045, the most relevant source was still inter-annual variability; while when considering the period 2081–2100, total variance was mostly explained by differences among climate models (46.6 %). Sampling variability around threshold and slope was a very important source of uncertainty, explaining 39.2 % of total variance for the period 2026–2045 and 40.3 % for the period 2081–2100. The model used in the population projections was not a major source of uncertainty, as indicated by the low value of the corresponding total-effect index.

**Table 3:** Total-effect index for each source of variability, for the time periods 1986–2005, 2026–2045 and 2081-2100

Source	1986–2005	2026–2045	2081–2100
Heat-mortality curve	27.8 %	39.3 %	40.3 %
Inter-annual variability	80.4 %	57.6 %	27.6 %
Climate model	-	44.6 %	46.6 %
Population model	-	0.9 %	6.8 %

## Discussion

The heat–mortality relationship estimated for Skopje for the sample period 2007–2011 is consistent with the results reported in the literature for other European cities. In particular, when maximum apparent temperature was used as exposure indicator, allowing comparison with published studies, we estimated a threshold equal to 30.6 °C and a variation above the threshold equal to 4.47 % (90 % CI: 2.56; 6.42). These values are similar to those obtained for the Mediterranean cities in the PHEWE study [23], in the EU CIRCE project [3], and for Lisbon and Porto [41].

During the baseline period 1986–2005, and assuming that the exposure–response functions stayed constant over time, we estimated that daily mean temperatures above the threshold were responsible on average for 58 deaths per year (slightly less than during the sample period 2007–2011), corresponding to an average AF of 3.3 %, that is close to the AFs estimated for Budapest and Turin during the 1990s by Baccini and colleagues [46]. Under the assumption that the heat–mortality relationship in the last 30 years was unchanged and equal to that estimated for the sample period 2007–2011, this result suggests that heat during the summer has been in the past, and is currently, an important public health problem in Skopje. An increasing impact of heat, given the set of assumptions used in this study, would be expected in the future. Considering all different combinations of climate models and population growth models, the average number of AD per year is expected to range from 95 to 125 for the time period 2026–2045, and from 186 to 366 for the time period 2081–2100, when AF could reach 13.2 %, under the maximal climate model.

In assessing the future impact of heat on mortality in Skopje, we accounted for several sources of uncertainty. While we used a Monte Carlo approach to account for sampling variability around the estimate of heat–mortality relationship [46], uncertainty related to future weather conditions was treated by generating annual temperature time series under different climate models and uncertainty related to population predictions was treated by using simple alternative population growth models. The contribution of each source of variability to the total variance of AD predictions was evaluated. Total variance was largely explained by differences among climate models. However, inter-annual variability of temperatures and sampling variability around threshold and slope estimates also had an important role, strongly impacting on the final variance. The contribution of the population projections to the total variance was small. Wu et al. [49] found a similar result when evaluating the future impact of heat waves on mortality in the eastern United States. However, it should be stressed that in our study the variability associated to population projections is strongly dependent on the specific growth models adopted. We cannot exclude that using different and more complex models, population projections could appear more critical to the final impacts, as has been reported elsewhere [19].

Our approach has several limitations. We assumed that the heat–mortality relationship does not change over time due to possible acclimatization/mitigation/adaptation of the city population, despite the decrease in negative impacts of heat during recent decades reported in the recent literature, possibly due to increasing availability of adaptation-oriented technologies, implementation of heat warning systems, and behavioural changes [17]. Moreover, we estimated this relationship on a limited number of years: from 2007 to 2011. On the one hand, this is an obvious limitation because a longer time series would have provided more stable results; on the other hand, by limiting the analysis to recent years, we have likely produced an estimate of the heat-mortality relationship with a higher chance of being valid in future time periods.

We focused on the effect of mean temperatures exceeding the estimated threshold, assuming a linear effect of heat above this threshold (on a logarithmic scale). We considered neither the possible additional effect of heat waves nor deviations from linearity for very high exposures [50]. We preferred this simple parametrization than a flexible modelling of the heat–mortality relationship for two main reasons: first, the boundary regions of the estimated curve can be very sensitive to outlying observations, so that for the highest temperatures the external validity of the curve could be compromised by the strong influence of a few observations; second, summarizing the relationship of interest by only two parameters (threshold and slope) permitted to implement the Monte Carlo approach in a simpler way. However, this choice has some consequences: mainly, we expect that the impacts can be underestimated for large exposures, leading to conservative results.

Our approach did not account for the inter-relation between population growth and changes in the urban landscape, which could result in significant alterations of urban temperatures and the urban heat island effect. In addition, future population size and climate conditions were assumed to be independent; while this may be a reasonable assumption for analysis at the local level, ideally, multifactor models like the Shared Socioeconomic Pathways should be used [51]. Moreover, we used simple deterministic population growth models, while evaluations of future population size arising from combination of population fertility, life expectancy and migration could be more accurate [20, 52, 53]. The United Nations [54] produced such predictions for Skopje (but only until 2030) whereby the city population is expected to increase in the coming years (despite the expected decrease of the national population). These predictions followed rates similar to those estimated up to 2030 by our models. A further limitation of the adopted population growth models is that they do not provide projections of the future age-structure of the city population. This limitation, coupled with the difficulty of obtaining stable estimates of the heat–mortality relationship by class of age, prevented us from performing age-stratified impact assessment.

In the presence of mortality displacement, a portion of deaths attributable to heat could be among very frail people with only a few weeks/days life expectancy. Our analysis does not capture this phenomenon, which could be very relevant. In the 15 cities participating in the PHEWE project, accounting for 30 day mortality displacement brought an overall impact reduction of 75 % [55].

Only one Representative Concentration Pathway climate scenario has been used. Ideally, other RCP’s should be used as well, but this requires much larger computational resources that what was available for this study. These results, based on RCP8.5, represent an upper bound for the temperatures (and hence the mortality, accounting for population increase and everything else being equal). However, it is important to stress that current emission trends thus far seem to continue to track along the trends of this scenario.

The daily urban temperatures for Skopje were obtained as unweighted means of the grid cell temperatures arising from the UrbClim model. In the absence of fine spatial projections of the population distribution within the city boundaries, and considering that people move from their residence during the day experiencing different exposure levels within the city, using a single urban exposure was an obvious choice. The validity of this choice is supported also by the high agreement between the daily mean temperatures in the ten districts of Skopje under all climate models and time periods (results not reported).

The study at hand assumes the urban form to be constant in time. As such, the mortality figures for the future time periods provide results for an unadapted city. Mortalities in well-adapted cities will probably be lower, as other studies have indicated that the negative effects of climate warming can be reduced by urban adaptation [56]. On the other hand, increased urbanization can increase the magnitude of the UHI-effect and the heat-related health effects in urban agglomerations [57, 58].

In addition, a more exhaustive evaluation of the impact of climate change in Skopje should also consider a possible reduction in cold-related mortality in the future, an effect observed for other geographical setting under various projections [59, 60].

Acknowledging these limitations, the importance of our results in the context of health adaptation against heat is clear. In the absence of adequate interventions and of significant acclimatization, a strong increase in heat-related mortality may be expected in Skopje. In light of warming projections in South East Europe, and pending further research, similar trends may occur in other urban areas in the Balkans. Regarding what constitutes “adequate interventions”, a recent review of the evidence suggests that heat-warning systems coupled with specific actions (health information plans, cooling centres, etc.) are effective in reducing health effects from heat [61–63]. An effective long-term heat–health prevention, however, would comprise not only the yearly implementation of these systems, but also a range of long-term measures, including adaptation in the built environment [64].

As of 2013, five countries in the Balkans (Croatia, the Republic of Moldova, Romania, Serbia and the former Yugoslav Republic of Macedonia) had plans in place to prevent and minimize health effects from heat [65]. However, when measured against a set of core elements for a heat–health action plan (HHAP) framework proposed by WHO [66], these plans showed uneven levels of implementation, particularly in the long-term measures pertaining to health systems preparedness and urban planning, as well as surveillance and evaluation [65]. Projections of increased impacts of heat in the medium and long-term (such as those in this article) strongly suggest a need for periodical re-evaluation and strengthening of long-term measures within the existing heat–health action plans. Moreover, heat–health action plans at the national or regional level can be supported and strengthened through additional local measures. While the choice of these measures may vary according to the local context and the availability of financial and human resources, there are crucial advantages in the involvement of local authorities in heat–health action, including effectiveness of outreach, fluid interaction with stakeholders, and clear competencies over urban planning [67].

## Conclusions

Heat is causing significant excess summer mortality in Skopje, and this effect may worsen under a likely climate change scenario. These results may help prompt health authorities in South East Europe and elsewhere towards adopting a long term perspective in managing the health effects of temperature in the context of a changing climate, as well as planning and implementing adequate adaptation measures in order to protect the health of residents from heat and heat waves.

The best available evidence must be used to evaluate such adaptation. In that context, city-level studies like the one presented in this paper can contribute to evidence-based decision support. More research is needed on city-specific impacts of heat on health in Europe, particularly in accession countries and other countries neighbouring the European Union.

### Ethics approval and consent to participate

Not applicable.

### Consent for publication

Not applicable.

### Availability of data and materials

The main dataset supporting the conclusions of this manuscript can be found and freely downloaded at http://www.ramses-cities.eu/results.

## Additional files

Additional file 1: Population Growth Models. (DOCX 19 kb) Additional file 2: Health impact assessment dataset. (DTA 91 kb)

## Abbreviations

AD: attributable deaths
AF: attributable fraction
AR5: fifth assessment report of the Intergovernmental Panel on Climate Change
CI: confidence interval
CIRCE: Climate Change and Impact Research: the Mediterranean Environment
CMIP5: Coupled Model Intercomparison Project 5
DiSIA: Department of Statistics, Informatics and Applications, University of Florence
ECMWF: European Centre for Medium-Range Weather Forecasts
EU: European Union
GCMs: global climate models
GEE: generalized estimating equations
HHAP: heat-health action plan
IPCC: intergovernmental panel on climate change
ISPO: Cancer Prevention and Research Institute
NACLIM: North Atlantic Climate Collaborative Project
NCDC: United States National Climatic Data Center
NDVI: Normalized Difference Vegetation Index
PHEWE: assessment and Prevention of acute Health Effects of Weather conditions in Europe
PM10: particulate matter less than 10 micrometers in diameter
RAMSES: Reconciling Adaptation, Mitigation and Sustainable Development for Cities
RCP: Representative Concentration Pathway scenario
UHI: urban heat island effect
VITO: Flemish Institute for Technological Research
WHO: World Health Organization
