# Supplementary material for: Projected heat-related mortality under climate change in the metropolitan area of Skopje
Source: BMC Public Health. 2016 May 16;16:407. doi: 10.1186/s12889-016-3077-y (PMC4868033; doi:10.1186/s12889-016-3077-y)
Supplement: Additional file 1: — Population Growth Models. (DOCX 19 kb) [file 12889_2016_3077_MOESM1_ESM.docx]

**Additional file 1 – Population Growth Models**

The exponential model for population growth assumes that each year population increases according to the same growth rate r:

|  | $P_{t}=P_{0}e^{rt}$ | (1) |
| --- | --- | --- |

On the other hand, the logistic model assumes a limit for the population size::

|  | $P_{t}=\frac{K}{1+Ae^{-kt}}$  $A=[K-P_{0}]/P_{0}$ | (2) |
| --- | --- | --- |

Where:

K is a the carrying capacity,

P_0_ is the initial population and

P_t_ is the population at time t

The growth rate of the exponential model was estimated by considering population at 2002 as initial population (P_2002_= 506,926) and comparing it with population size after ten years (P_2012_= 531,524):

|  | $e^{r}={(P_{2012}/P_{2002})}^{0.1}$ | (3) |
| --- | --- | --- |

Regarding the logistic model, *A* and *k* were estimated according to the following equations involving the carrying capacity parameter K:

|  | $A=(K-P_{2002})/P_{2002}$  $k=-[\ln\left( K-P_{2012} \right)-\ln\left( AP_{2012} \right)]/10$ | (4) |
| --- | --- | --- |
